# Brain-predicted age in Down syndrome is associated with beta amyloid deposition and cognitive decline

**DOI:** 10.1016/j.neurobiolaging.2017.04.006

**Published:** 2017-08

**Authors:** James H. Cole, Tiina Annus, Liam R. Wilson, Ridhaa Remtulla, Young T. Hong, Tim D. Fryer, Julio Acosta-Cabronero, Arturo Cardenas-Blanco, Robert Smith, David K. Menon, Shahid H. Zaman, Peter J. Nestor, Anthony J. Holland

**Affiliations:** a Computational, Cognitive & Clinical Neuroimaging Laboratory (C3NL), Division of Brain Sciences, Imperial College London, London, UK; b Cambridge Intellectual and Developmental Disabilities Research Group, Department of Psychiatry, University of Cambridge, Cambridge, UK; c Medicine School, University of Birmingham, Birmingham, UK; d Wolfson Brain Imaging Centre, University of Cambridge, Cambridge, UK; e German Center for Neurodegenerative Diseases (DZNE), Magdeburg, Germany; f Division of Anaesthesia, Department of Medicine, University of Cambridge, Cambridge, UK

**Keywords:** Down syndrome, Brain aging, Amyloid PET, MRI, Machine learning, Cognitive decline

## Abstract

Individuals with Down syndrome (DS) are more likely to experience earlier onset of multiple facets of physiological aging. This includes brain atrophy, beta amyloid deposition, cognitive decline, and Alzheimer's disease—factors indicative of brain aging. Here, we employed a machine learning approach, using structural neuroimaging data to predict age (i.e., brain-predicted age) in people with DS (N = 46) and typically developing controls (N = 30). Chronological age was then subtracted from brain-predicted age to generate a brain-predicted age difference (brain-PAD) score. DS participants also underwent [^11^C]-PiB positron emission tomography (PET) scans to index the levels of cerebral beta amyloid deposition, and cognitive assessment. Mean brain-PAD in DS participants' was +2.49 years, significantly greater than controls (*p* < 0.001). The variability in brain-PAD was associated with the presence and the magnitude of PiB-binding and levels of cognitive performance. Our study indicates that DS is associated with premature structural brain aging, and that age-related alterations in brain structure are associated with individual differences in the rate of beta amyloid deposition and cognitive impairment.

## 1 Introduction

Down syndrome (DS), the result of trisomy 21, results in intellectual disability and a set of characteristic physiological and behavioral traits. In middle adulthood, people with DS also commonly experience clinical symptoms that are normally associated with older age (Covelli et al., 2016, Devenny et al., 2005). Whether DS truly results in an acceleration to all aspects of the aging process is controversial (Zigman, 2013). Nevertheless, skin wrinkles, gray hair and alopecia, visual and auditory decline, hypogonadism, hypothyroidism, osteoporosis, and the menopause all occur considerably earlier in people with DS (Covelli et al., 2016, Zigman, 2013). Beside these physiological manifestations of aging, evidence also suggests that people with DS experience premature brain aging. Cognitive decline and subsequent Alzheimer's Disease (AD) occur more frequently and at an earlier age in DS (Devenny et al., 2000, Holland et al., 1998, Margallo-Lana et al., 2007, Wisniewski et al., 1985). In addition, postmortem and in vivo studies show increased cerebral beta amyloid deposition, neurofibrillary tau tangles, brain atrophy, and white matter lesions in DS (Annus et al., 2016, Head et al., 2016, Lao et al., 2016, Mann and Esiri, 1989, Nelson et al., 2011, Roth et al., 1996, Wisniewski et al., 1985). All these changes have been associated with the typically aging brain, albeit at an older age (Braskie et al., 2010, Fjell et al., 2009, Jack et al., 2014, Rodrigue et al., 2012, Wardlaw et al., 2013). However, despite this increased prevalence of “age-like” changes observed in people with DS, the onset of the symptoms of brain aging and subsequent trajectories of decline show marked variability (Oliver et al., 1998). While the biological mechanisms underlying these brain changes in DS are likely distinct from normal aging, their manifestation can still be assessed using the same techniques, such as neuroimaging. Therefore, understanding the relationships among beta amyloid deposition, brain structure, and cognitive decline should help capture these individual differences and allow better prediction of health outcomes.

To measure how brain structure changes with aging, multivariate machine learning methods have been developed that allow accurate prediction of chronological age using neuroimaging data (Dosenbach et al., 2010, Franke et al., 2010, Mwangi et al., 2013). This has demonstrated that neuroimaging data can be used to generate an index that quantifies age-related changes to brain volume, which we refer to as “brain-predicted age.” Accordingly, brain-predicted age, or equivalent, has been used to demonstrate that environmental factors are associated with a person's brain appearing younger or older than would be expected for their chronological age. Deleterious influences on brain-predicted age include traumatic brain injury (Cole et al., 2015), obesity (Ronan et al., 2016), schizophrenia (Koutsouleris et al., 2014, Schnack et al., 2016), HIV infection (Cole et al., 2017), diabetes (Franke et al., 2013), mild cognitive impairment, and AD (Franke and Gaser, 2012, Gaser et al., 2013). Conversely, the protective effects of physical exercise, education, and meditation (Luders et al., 2016, Steffener et al., 2016) have also been reported, indicating that brain-predicted age can deviate outside of the context of atrophy due to brain diseases. The variability in brain-predicted age appears to be a useful way of quantifying individual differences in structural age-related changes to the brain in both disease and the general population.

Here, we sought to establish if people with DS show evidence of premature brain aging, using neuroimaging to measure brain-predicted age. Furthermore, we considered whether levels of beta amyloid deposition (according to positron emission tomography [PET] imaging), cognitive decline, and the manifestation of dementia relate to the levels of “age-like” changes to brain structure in DS.

## 2 Materials and methods

### 2.1 Participants

The study included N = 46 adults with DS (mean age = 42.30 ± 8.73 years, 25 males/21 females) and N = 30 typically developing adults as a control group (mean age = 46.23 ± 9.75, 16 males/14 females). All 76 individuals underwent MRI scans, while the DS group also underwent PET scans. Data on these individuals have been previously reported (Annus et al., 2016). All DS participants have previously received a clinical diagnosis of DS based on having the characteristic phenotype, while a proportion (N = 33) were karyotyped to confirm the presence of trisomy 21. Participants were screened to ensure that they had no contraindications to MRI and PET scanning. Ethical approval for the study was granted by the National Research Ethics Committee of East of England and the Administration of Radioactive Substances Advisory Committee (ARSAC). Written consent was obtained from all adults with DS with the capacity to consent. For participants lacking the capacity to consent, the procedures set out in the England and Wales Mental Capacity Act (2005) were followed. These 76 individuals comprised the brain-predicted age test set.

To define a multivariate model of healthy brain structure across the lifespan, data were collated from publicly available sources. This included N = 2001 typically developing, healthy individuals (mean age = 36.95 ± 18.12, age range 18–90 years, 1016 males/985 females), who comprised the brain-predicted age training set (see Supplementary Table 1). All training set participants were screened locally to exclude individuals with major neurologic or psychiatric diagnoses, a history of head trauma or major physical health problems, as per local study protocols. Each contributing study was granted ethical approval for data collection and subsequent data sharing. Informed consent was obtained at each local study site in accordance with local guidelines.

### 2.2 Clinical and neuropsychological assessment

All DS participants were assessed for dementia using an informant interview; the Cambridge Examination for Mental Disorders of the Elderly-DS version (CAMDEX-DS), adapted from the original CAMDEX for diagnosing dementia specifically in DS (Ball et al., 2004). These informant interviews were conducted by trained researchers at the University of Cambridge. Subsequently, an experienced clinician (SHZ), who was blinded to participant identity, used interview transcripts to assign DS participants to 1 of the 3 discrete categories: cognitively stable, cognitive decline, or dementia. Classification of dementia was in line with established criteria (International Classification of Disease 10). Cognitive decline was defined as evidence of cognitive impairment in one or more cognitive domains without fulfilling the full criteria for dementia. As part of the CAMDEX, all participants in the DS group underwent the Cambridge Cognitive Examination (CAMCOG; Holland et al., 1998). The CAMCOG collates functioning across a range of cognitive domains and generates a continuous measure of cognitive function, where higher scores indicate higher levels of cognitive performance. Three DS participants were unable to complete the CAMCOG assessment, due to having advanced symptoms of dementia.

### 2.3 Apolipoprotein E genotyping

DS participants supplied blood samples that were processed to determine the genotypes for apolipoprotein E (APOE). Peripheral blood samples were stored in EDTA tubes and the DNA extracted using standard methods. The DNA was genotyped for ApoE using primer pairs to amplify by polymerase chain reaction (PCR) the region containing the Arg/Cys polymorphism at codons 112 and 158 of the APOE gene. Standard PCR was performed using PCR mix (Bioline) using Taq polymerase to unambiguous typing of all homozygotic and heterozygotic isoform combinations. For analysis purposes, APOE genotype was used to define a binary categorization of each participant as either an e4 carrier or not an e4 carrier. APOE data were missing for N = 6 DS participants, from whom blood samples could not be collected.

### 2.4 Neuroimaging data acquisition

Full details of the PET acquisition protocol have been previously reported (Annus et al., 2016). In brief, PET data were acquired using a GE Advance scanner (General Electric Medical Systems, Milwaukee, WI, USA) to measure the levels of [^11^C]-Pittsburgh compound B (PiB) uptake across the brain. [^11^C]-PiB was injected as a bolus via a catheter and data were acquired for 90 minutes after injection. Fifty-eight frames were acquired as follows: 18 × 5 seconds, 6 × 15 seconds, 10 × 30 seconds, 7 × 60 seconds, 4 × 150 seconds, and 13 × 300 seconds. Sinogram data were then reconstructed, resulting in a voxel size of 2.34 × 2.34 × 4.25 mm. Visual inspection of the PET data was conducted to ensure that there were no major head movements or other artifacts present.

Structural images were T1-weighted MRI scans, acquired for the DS and control groups using the same Siemens Verio 3T scanner (Siemens AG, Erlangen, Germany). A magnetization-prepared rapid gradient echo (MPRAGE) sequence was used, with the following parameters: TR = 2300 ms, TE = 2.98 ms, TI = 900 ms, flip angle = 9°, and matrix dimension = 256 × 240. The protocol acquired 176 axial slices of 1 mm thickness, resulting in voxels of 1 mm^3^. Parallel acceleration was not enabled.

High-resolution T1-weighted data were also used from the training dataset, collated from previous studies. All training data were acquired at either 1.5T or 3T using standard T1-weighted sequences (e.g., MPRAGE, SPGR, T1-FFE).

### 2.5 PiB binding analysis

To measure [^11^C]-PiB uptake levels across the brain, nondisplaceable binding potential (BP_ND_) was calculated for different cerebral regions of interest (ROIs). This process involved 2 stages: the delineation of ROIs using T1-MRI and then the extraction of mean PiB BP_ND_ levels for each ROI from the PET data (as per Annus et al., 2016). To delineate cortical ROIs, a customized Brodmann atlas, registered to the Colin27 T1 template, was warped to a study-specific T1 template. This process was achieved using the Advanced Normalisation Tools (ANTS; Avants et al., 2011) and involved skull-stripping, affine global registration, and an iterative nonlinear local registration procedure to generate the study-specific template and register the atlas to the template. The Colin27-Brodmann atlas was then resampled to the study template space using nearest neighbor interpolation. Subcortical ROIs were also included, derived using FSL FIRST (Patenaude et al., 2011), resulting in a total of 30 ROIs. FIRST analysis was conducted in participant native space, before being resampled into study template space, again using nearest neighbor interpolation for discrete ROI images. To reduce the influence of partial volume effects (PVE), T1 images were segmented (i.e., tissue classified) using SPM12 (University College London, London, UK) to generate gray matter (GM), white matter (WM), and cerebrospinal fluid (CSF) probability images. These images were also warped to the study-specific templates using the ANTS transformations calculated as above.

PET data were also normalized to the study-specific template. This involved initially realigning the dynamic PET time-series and then averaging them across time using SPM12. These mean images were then rigidly registered to their corresponding native T1 images (which had been bias-field corrected), using ANTS. Then, by combining together the PET-T1 native and T1-study template transformations, PET images were normalized to the study template using a single resampling step, via trilinear interpolation.

Next, regional PiB BP_ND_ values were calculated, using PET data, cortical and subcortical ROIs, and tissue probability masks, all in study template space. For each ROI, thresholded at ≥65% GM probability, time-activity curves (TACs) were extracted from the normalized PET data. Tissue-input kinetic modeling was then carried out, using the superior cerebellum as a reference region (thresholded to ≥90% GM probability). As a further step to reduce partial volume effects, each TAC was divided by 1-CSF probability value in each voxel. The final BP_ND_ per ROI was then calculated using a basis function version of the simplified reference tissue model (Gunn et al., 1997).

To ascertain whether an individual's PET data indicated that they had abnormal levels of PiB binding (i.e., PiB-status), indicative of fibrillary beta amyloid deposition, our previously outlined procedure was used (Annus et al., 2016). This entailed defining a bimodal distribution of striatal (i.e., caudate and putamen) PiB BP_ND_ levels. Individuals with striatal PiB BP_ND_ <1 standard deviation (SD) from 0 were defined as PiB-negative, while individuals with striatal PiB BP_ND_ ≥1 SD from 0 were defined as PiB-positive. Subsequently, regional PiB BP_ND_ was defined as normal or abnormal per participant, based on the distribution of PiB BP_ND_ for a given region in the PiB-negative group. Abnormal PiB BP_ND_ was defined as ≥2 SDs from the PiB-negative group mean. Finally, the total number of ROIs defined as abnormal was summed per participant. Notably, individuals in the PiB-negative group did now show any evidence of abnormal PiB BP_ND_ in nonstriatal ROIs. In addition to classifying ROIs based on PiB BP_ND_, the mean PiB BP_ND_ across all cortical ROIs was calculated.

### 2.6 Brain-predicted age calculation

**Fig. 1:**
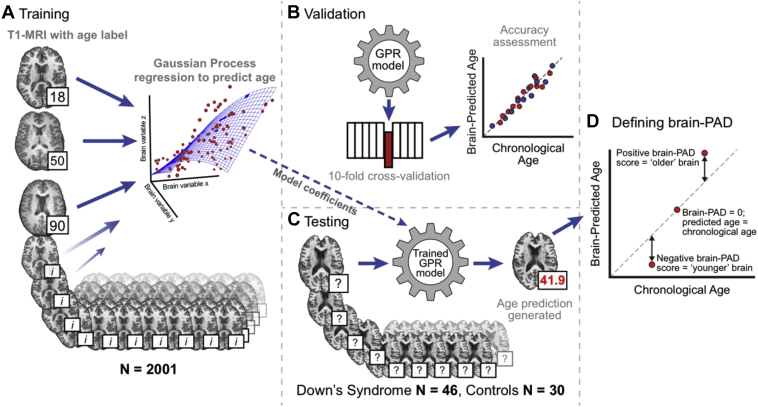
Overview of the brain-predicted age analysis pipeline. Illustration of the methods used to generate brain-predicted ages. 3D T1-weighted MRI scans were segmented into gray matter (GM) and white matter (WM) before being normalized to common space using nonlinear image registration. Normalized GM and WM images were concatenated and converted into vectors for each participant. These vectors were then projected into an N × N similarity matrix based on vector dot-products. (A) Once in similarity matrix form, the training participants' data were used as predictors in a Gaussian Processes regression model with age as the outcome variable. (B) Model accuracy was assessed in a 10-fold cross-validation procedure, comparing brain-predicted age with original chronological age labels. (C) Model coefficients learned during training were then applied to the data from DS participants and controls to generate brain-predicted ages. (D) A metric to summarize the variation in brain-predicted age was defined; the brain-predicted age difference. Abbreviation: brain-PAD, brain-predicted age difference.

An overview of the brain-predicted age calculation procedure is presented in Fig. 1. All structural images were preprocessed using SPM12. Images were bias corrected and segmented into GM, WM, and CSF using SPM Segment. Visual quality control was carried out at this stage to ensure the accuracy of image segmentation; all images were included for both groups. Segmented images for GM and WM were then nonlinearly registered to a custom template, based on the training dataset, using SPM DARTEL (Ashburner, 2007). Finally, images were affine registered to MNI152 space (voxel size = 1.5 mm^3^) and resampled using modulation to retain volumetric information and smoothed with a 4-mm full-width half-maximum Gaussian kernel. Summary measures of brain volumes were also generated for GM, WM, CSF, and intracranial volume (ICV).

Brain-predicted ages were generated as previously outlined (Cole et al., 2015), using the Pattern Recognition for Neuroimaging Toolbox (PRoNTo v2.0, www.mlnl.cs.ucl.ac.uk/pronto) software package. First, a model of healthy brain aging was defined using brain volumetric maps from the training dataset (N = 2001) as follows: Spatially normalized images were converted to vectors and the resulting GM and WM vectors were concatenated for each individual. A linear kernel representation of these data was derived by calculating an *N* × *N* similarity matrix, where each point in the matrix was the dot product of 2 participants' image vectors. A Gaussian Processes regression model was then defined, with chronological age as the dependent variable and 3-dimensional brain volumetric image data (in similarity matrix form) as the independent variables.

Predictions for all training participants were generated using 10-fold cross-validation, whereby the data were randomly divided into 10 folds, each comprising 10% of the participants. The model was then retrained using 9 folds of the data and age predictions were made for data in the “left-out” fold. This procedure was iterated so that all folds were left out in turn, resulting in unbiased (i.e., independent) age predictions for each participant. Model accuracy was expressed as the correlation between age and brain-predicted age (Pearson's *r*), total variance explained (R^2^), mean absolute error (MAE), and root mean squared error (RMSE). Statistical significance of this model was assessed using permutation testing (n = 1000).

Next, the coefficients from the full training model (N = 2001) were applied to the test data (i.e., DS participants and controls), to generate unbiased brain-predicted ages. Finally, brain-predicted age difference (brain-PAD) scores were calculated for each individual in the DS and control groups by subtracting chronological age from brain-predicted age. Hence, a positive brain-PAD score indicates that the individual's brain is predicted to be “older” than their chronological age. Brain-PAD scores were subsequently used for further analysis to index relative structural brain aging.

### 2.7 Statistical analysis

Using brain-PAD values, further statistical analysis was conducted to compare the experimental groups and assess the relationships between variables. Data were assessed for normality and parametric tests deemed appropriate for use. Fisher's exact tests were used to compare the frequencies of categorical variables, Pearson's correlations were used to relate continuous variables and t-tests were used to compare means between groups. A linear regression model was run with brain-PAD as the outcome variable and group as the predictor, to compare brain-PAD between DS participants and controls. Linear regression was also used to compare ICV between groups. To establish whether brain-PAD predicted characteristics of the DS participants, logistic regressions for categorical outcome variable (PiB-status, CAMDEX classification) and linear regressions for continuous outcome variable (mean BPND, number of PiB-abnormal ROIs, and CAMCOG score) models were run, with age as a covariate. All statistical analysis of brain-PAD was conducted using R v3.3 (R Core Team, 2015).

## 3 Results

### 3.1 Cohort description

Compared to controls, DS participants were trending toward being younger (Table 1, *p* = 0.08), while the ratio of males to females was similar between groups (*p* = 0.96). DS participants who were positive for PiB-binding were older than PiB-negative counterparts (*p* < 0.001) and had higher rates of cognitive decline and dementia according to CAMDEX classification (*p* < 0.001), though similar levels of cognitive performance, according to the CAMCOG assessment (*p* = 0.33). There was no relationship between PiB-status and sex (*p* = 0.78).

**Table 1:** Characteristics of Down syndrome participants and controls

Characteristic	DS (all)	DS PiB-positive	DS PiB-negative	Controls
N	46	19	27	30
Mean age (y)	42.3 (8.73)	49.68 (6.45)	37.11 (5.95)	46.23 (9.75)
Age range (y)	28–65	39–65	28–48	30–64
Sex (male/female)	25/21	11/8	14/13	16/14
CAMDEX classification (stable, declining, dementia)	31/6/9	7/5/7	24/1/2	-
CAMCOG score	74.37 (20.01)	70.19 (22.98)	76.85 (18.03)	-
APOE genotype (e2/e3, e2/e4, e3/e3, e3/e4/missing)	8/2/20/10/6	4/1/6/5/3	4/1/14/5/3	-

Values presented in the table are either N, or in mean (standard deviation) form.

### 3.2 Age can be accurately predicted using neuroimaging

The machine learning model accurately predicted chronological age in the training dataset, based on T1-weighted MRI. Ten-fold cross-validation resulted in a correlation between brain-predicted age and chronological age of *r* = 0.94 (*p* = 0.001, corrected after 1000 permutations) and explained 88% of the variance (R^2^). The MAE of prediction = 5.02 years and the RMSE = 6.31 years. This training stage validated our model of brain-predicted age for use in predicting age with neuroimaging data in the test set, comprising DS participants and controls.

### 3.3 Down syndrome is associated with increased brain-predicted age difference

**Fig. 2:**
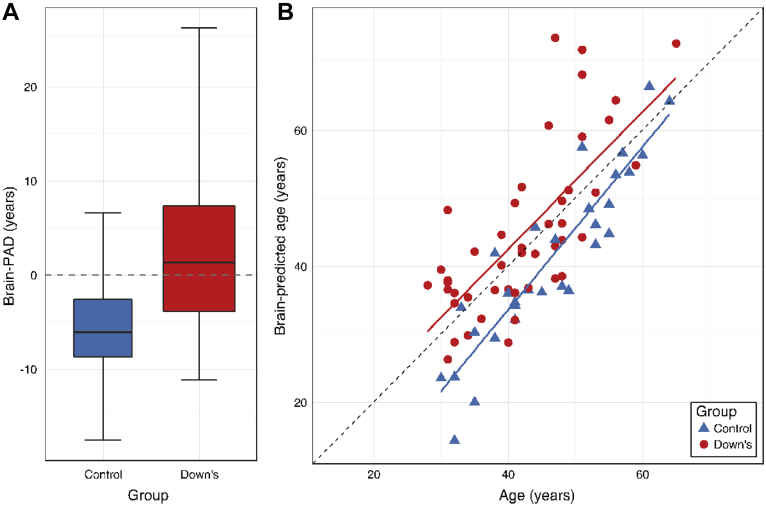
Brain-predicted age in individuals with Down syndrome (DS) and controls. (A) Box plot of brain-PAD (years) according to group, showing DS participants (red box) and controls (blue box). Whiskers (i.e., bars) on the box plots represent the absolute range of data points for each group. (B) Scatterplot of age (x-axis) and brain-predicted age (y-axis) indicates DS participants (red circles) and controls (blue controls). Plotted are linear regression lines representing a linear fit of age regressed onto brain-predicted each, colored according to group (DS = red line, control = blue line). Abbreviation: brain-PAD, brain-predicted age difference. (For interpretation of the references to color in this figure legend, the reader is referred to the Web version of this article.)

Brain-PAD in DS participants was significantly greater than controls (*b* = 7.69 [95% confidence intervals = 4.3, 11.1], SE = 1.72, *t* = 4.46, *p* < 0.001, Fig. 2A). Mean brain-PAD in the DS group was 2.49 years (SD = 8.25), while in the control group mean brain-PAD was −5.20 years (SD = 5.67). Brain-PAD in the DS group was significantly greater than the training set mean (*t* = 2.04, *p* = 0.04). Brain-predicted age was significantly correlated with chronological age in both DS participants (*r* = 0.72, *p* < 0.001) and in controls (*r* = 0.91, *p* < 0.001, Fig. 2B). There were no differences in prediction accuracy between groups (*p* = 0.91), with the MAE in DS = 6.65 (SD = 8.20) years and in controls MAE = 6.46 (SD = 5.67) years. The RMSE = 8.53 in DS and 7.62 in controls.

As head size has been shown to differ between individuals with DS and typically developing individuals, we also investigated the influence of ICV on brain-PAD. Indeed, DS participants did have reduced ICV relative to controls (DS mean = 1.19L [SD = 0.11], control mean = 1.42L [SD = 0.14], *b* = −0.02, SE = 0.03, *t* = −7.89, *p* < 0.001). However, ICV was not significantly correlated with brain-PAD in the DS group (*r* = −0.04, *p* = 0.77) or the control group (*r* = 0.09, *p* = 0.62). There were no sex differences in brain-PAD in either the DS group (*t* = 0.66, *p* = 0.51) or the control group (*t* = 1.00, *p* = 0.32). APOE genotype did not significantly influence brain-PAD (*t* = −1.63, *p* = 0.11).

### 3.4 Brain-predicted age increases are associated with increased PiB binding potential

**Fig. 3:**
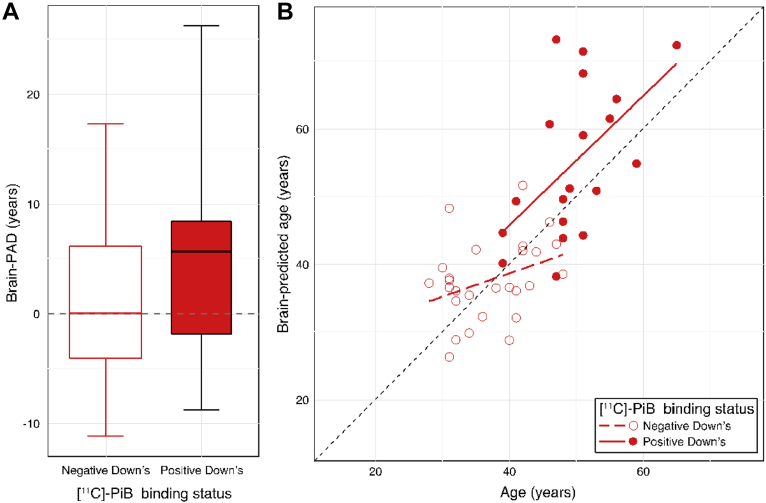
Brain-predicted age in individuals with Down syndrome (DS), according to [^11^C]-PiB status. (A) Box plot of brain-PAD (years) according to [^11^C]-PiB status in DS participants. PiB-positive (red box) and PiB-negative (white box). Whiskers (i.e., bars) on the box plots represent the absolute range of data points for each group. (B) Scatterplot of age (x-axis) and brain-predicted age (y-axis) indicate PiB-positive DS participants (filled red circles) and PiB-negative DS participants (open red circles). Plotted are linear regression lines representing a linear fit of age regressed onto brain-predicted each, colored according to group (PiB-positive = solid red line, PiB-negative = dashed red line). Abbreviations: [^11^C]-PiB, [^11^C]-Pittsburgh compound B; brain-PAD, brain-predicted age difference. (For interpretation of the references to color in this figure legend, the reader is referred to the Web version of this article.)

Within the DS group, 19 individuals were classified as PiB positive based on PET data, whereas 27 individuals showed no evidence of abnormal PiB binding (PiB negative). The mean cortical PiB BP_ND_ across PiB-positive individuals = 0.36 ± 0.22, while the median number of ROIs showing abnormal PiB levels in these individuals was 28 (range 1–30). Brain-PAD significantly predicted PiB-status in DS participants (*b* = 0.20 [95% confidence intervals = 0.03, 0.44], SE = 0.10, z = 1.98, *p* = 0.048), as did chronological age (*b* = 0.42, SE = 0.14, z = 3.11, *p* = 0.002), in a logistic regression model. The group mean brain-PAD scores were 5.29 ± 9.41 years in PiB-positive DS participants and 0.52 ± 6.84 years in PiB-negative DS participants (Fig. 3A). Brain-predicted age was significantly correlated with chronological age in PiB-positive individuals (*r* = 0.54, *p* = 0.02), though this relationship was only borderline in PiB-negative DS participants (*r* = 0.32, *p* = 0.08, Fig. 3B). Brain-PAD significantly predicted both mean cortical PiB BP_ND_ (*b* = 0.008, SE = 0.003, *t* = 2.78, *p* = 0.001) and the number of ROIs showing abnormal PiB levels (*b* = 0.46, SE = 0.14, *t* = 3.40, *p* = 0.001), when covarying for chronological age (which was also significantly related to both quantitative PiB measures, *p* < 0.05) in DS participants.

### 3.5 Brain-predicted age increases are related to poorer cognitive performance in PiB-positive individuals

**Fig. 4:**
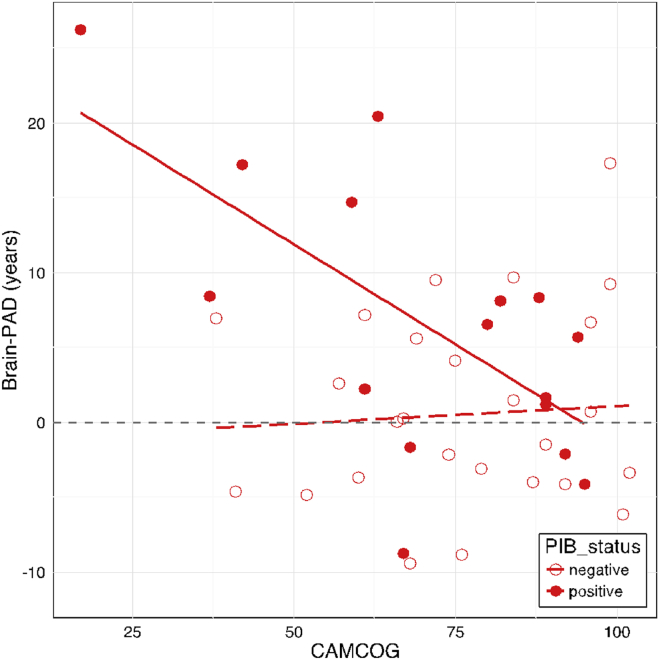
Cambridge Cognitive Battery (CAMCOG) performance relates to brain-PAD, according to [11C]-PiB status. Scatterplot of CAMCOG score (x-axis) against brain-PAD (y-axis) in DS participants indicate PiB-positive individuals (filled red circles) and PiB-negative individuals (open red circles). Plotted are linear fit lines of CAMCOG score regressed onto brain-PAD for each group (PiB-positive: solid line; PiB-negative: dashed line), to illustrate the interaction between brain-PAD and PiB-status in predicting CAMCOG score. Abbreviations: [^11^C]-PiB, [^11^C]-Pittsburgh compound B; brain-PAD, brain-predicted age difference. (For interpretation of the references to color in this figure legend, the reader is referred to the Web version of this article.)

When considering all DS participants together, there was a significant effect of brain-PAD on CAMCOG score (b = −0.80, SE = 0.35, *t* = −2.24, *p* = 0.03), whereby higher brain-PAD was associated with worse performance on the CAMCOG assessment. However, when considering PiB status in the model, there was a significant interaction between brain-PAD and PiB status (b = −1.75, SE = 0.72, *t* = −2.42, *p* = 0.02), when predicting CAMCOG score (Fig. 4). This indicates that in PiB-positive DS participants there is a strong negative relationship between brain-PAD and CAMCOG score, while in PiB-negative DS participants, there is no such relationship. This was despite there being no significant difference in CAMCOG score based on PiB status, as noted above. Age was not included as a covariate in these analyses as age was not related to CAMCOG score (*p* = 0.33). Ordinal logistic regression analysis indicated that brain-PAD score did not significantly predict CAMDEX classification, either with or without PiB status as an additional covariate (*p* = 0.73; *p* = 0.72). Interestingly, when excluding DS individuals with declining or dementia ratings from the CAMDEX (i.e., limiting the analysis to cognitively stable individuals, N = 31), there was still a significant effect of group on brain-PAD (b = 7.34, SE = 1.75, t = 4.19, *p* < 0.001). We then further investigated the relationship between PiB status and CAMDEX classification, though we collapsed the CAMDEX into a binary classification of stable versus not stable (i.e., declining or dementia). There was a significant relationship between PiB-status and CAMDEX (*p* < 0.001), as DS individuals were more likely to be stable if there were PiB-negative, and more likely to be declining if they were PiB-positive (see Supplementary Table 2). However, there was no main effect of these subgroups on brain-PAD (*p* = 0.23).

Finally, we considered how the magnitude of PiB-binding (measured by mean BP_ND_) and brain-PAD related to cognitive impairment (i.e., CAMCOG score). A linear regression model including mean BP_ND_ and brain-PAD as predictor variables and CAMCOG score as the outcome was significant (*p* = 0.025) and explained (R^2^ =) 12.6% of the variance in CAMCOG.

## 4 Discussion

Individuals with DS showed increased brain-PAD scores, indicating that the three-dimensional patterns of brain volume associated with DS resembled that of healthy individuals on average 2.49 years older. Moreover, the effect of DS on brain-PAD was an adjusted increase of 7.69 years, compared to scanner matched, typically developing controls. There was considerable variability observed in brain-PAD in DS participants. Notably, this variability related to measures of amyloid deposition, indexed by [^11^C]-PiB PET imaging and to cognitive decline, according to CAMCOG assessment. The relationship between brain-PAD and CAMCOG score was observed only in individuals with evidence of amyloid deposition, indicating that changes in brain structure and cognitive performance may be linked to the deleterious build-up of amyloid in DS.

This is the first application of a neuroimaging-based “brain-predicted age” index in people with DS. The observed increased brain-PAD supports the idea that the long-term consequences of DS include premature “age-like” changes to the structure of the brain. Previously, studies have shown lower brain volumes or abnormal cortical thickness in DS (Koran et al., 2014, Mullins et al., 2013, Pinter et al., 2001, Romano et al., 2016, Teipel et al., 2004) as well as a correlation between age and brain volume in individuals with DS, not seen in controls (Beacher et al., 2010, Krasuski et al., 2002). While these results have been used to indirectly infer the presence of “accelerated” brain aging in DS, our machine learning method provides a more direct approach of quantifying age-related changes to brain structure, by way of reference to a large lifespan training sample. Moreover, this technique captures voxel-wise variation in brain volumes, incorporating higher resolution data into the prediction, and is more appropriate for making individualized predictions, rather than relying on group-average trends. Crucially, however, cross-sectional analysis can only suggest “premature” or “accentuated” brain aging, which is a limitation of the current analysis. Longitudinal studies are necessary to determine whether these age-like changes to brain structure are static or are accelerating over time.

Various mechanisms could underlie the increase in brain-PAD observed in DS. One candidate is the triplication of the amyloid precursor protein (APP) gene, found on chromosome 21, which results in increased levels of APP (Rumble et al., 1989). As APP is necessary for amyloid protein production, its overexpression may result in increased amyloid levels and subsequent development of neuritic plaques, a key risk factor for AD. Hence, APP triplication may explain the earlier onset and higher prevalence of AD in DS. In our study, PET-derived measures of the magnitude of fibrillary beta amyloid deposition were related to brain-PAD in DS participants, when accounting for the age dependence of PiB levels (Hartley et al., 2014). Conversely, brain-PAD did not relate to CAMDEX classification, though interestingly, a model containing brain-PAD and mean PiB-binding significantly predicted CAMCOG scores. This highlights the convergence of amyloid deposition, structural brain changes, and cognitive impairment in DS. While determining causality from these cross-sections is not possible, our findings suggest that the accumulation of fibrillary beta amyloid may be a precursor to the loss of brain tissue volume and cognitive changes, with dementia symptoms manifesting later in the timeline of neurodegenerative processes.

Other factors that potentially explain premature brain aging in DS include the triplication of other genes on chromosome 21, such as SOD-1 and SLC5A3, involved in response to oxidative stress and in moderating cerebral myoinositol levels, respectively (Beacher et al., 2010). Evidence also suggests that mitochondrial dysfunction occurs in DS in both peripheral and central cells (Phillips et al., 2013, Tiano and Busciglio, 2011). Maintenance of mitochondrial functions, particularly the production of ATP, is crucial to many aspects of cellular function, and mitochondrial changes have been implicated in aspects of aging (reviewed by Lopez-Otin et al., 2013). Plausibly, mitochondrial dysfunction in neurons and astrocytes caused by trisomy 21 could impact on the metabolism of APP (Busciglio et al., 2002) and result in a cascade of downstream effects that prematurely age brain structure in DS.

From an environmental perspective, physical health and dietary factors, as evidenced by the increased rates of obesity (Melville et al., 2005), may have indirect effects on physiological “wear-and-tear.” This could contribute to multiple aspects of aging in DS, including the brain. Cerebrovascular factors, such as cerebral amyloid angiopathy and microhemorrhages, perhaps as a result of persistent neuroinflammation (Wilcock et al., 2016), may also be involved. Such factors are thought to contribute to brain atrophy outside of the context of DS (Chowdhury et al., 2011, Muller et al., 2011); however, they are unlikely to be entirely independent of the genetic and environmental issues discussed above. A cluster of inter-related risk factors appears to be present in DS, negatively impacting the neural milieu and hence increasing the rate of tissue volume loss normally associated with aging.

Ours is not the first study to assess the tissue-specific measure of aging in DS. Previously, shortened lymphocyte telomere length has been reported in people with DS (Jenkins et al., 2006, Vaziri et al., 1993) and shorter telomeres have been associated with cognitive decline and dementia status in DS (Jenkins et al., 2010, Jenkins et al., 2016). Indices of age-associated oxidative stress have also been reported as elevated in DS (Jovanovic et al., 1998). Recently, Horvath et al., (2015) reported “accelerated” epigenetic aging in people with DS, using a multivariate method to predict age based on DNA methylation status in blood, buccal cells, and brain tissue. This indicates that premature age-like changes are occurring at a molecular level in individuals with DS, of which the reduced brain volume detected by brain-predicted age may be a macroscopic manifestation. Interestingly, the “residual” approach adopted by Horvath et al., which essentially sets the control group mean to zero, resulted in an average effect of DS equivalent to 6.6 years accelerated aging. Taking the same approach here, we see an effect of a similar magnitude, 7.4 years of added aging. In future studies, it could prove informative to combined multiple aging biomarkers (e.g., telomere length, epigenetic clock, and brain-predicted age) to assess whether predictions of cognitive decline and dementia risk can be improved.

A potential criticism of neuroimaging models of brain-predicted age is that results could be driven by deviation from normality, as opposed to divergence along a specifically age-related trajectory. While such deviations may occur in neurologic conditions, such as stroke or encephalopathy, this is not the case in DS. Brain atrophy in DS is likely to be gradual and cumulative, caused by factors such as increased amyloid deposition or secondary effects of exposure to abnormal neurodevelopment, rather than resulting from a one-off insult. While DS brains have been shown to be abnormal in size and morphology (Annus et al., 2017, Wang, 1996), brain-PAD scores did not correlate with ICV, nor does ICV correlate with brain-PAD in healthy individuals, so global size differences do not appear to be driving the results. Distinctive brain morphology in DS also did not seem to influence our findings as we observed no differences in the MAE of age prediction in the DS and control groups; age prediction accuracy was not hindered by any morphologic features of DS. While, in general, it can be difficult to distinguish between aging effects and disease effects in conditions such as DS, the fact that increased brain-PAD was seen when limiting the analysis to cognitively stable (as per CAMDEX classification) participants, supports the idea that it is not manifest disease that is driving age-related changes to brain structure.

Our study has some strengths and limitations. The use of a large independent healthy training dataset (N = 2001), on which the brain-predicted age values were based, allowed us to put brain aging in DS in context of what is expected in during healthy aging. However, one limitation of acquiring these data from various public sources is that we have inadequate demographic or behavioral data from which to quantify the characteristics of these individuals, other than that they were screened to be in good general and neurologic health. The use of multimodal neuroimaging (i.e., MRI and PET) to provide data on age-related brain changes in DS from independent sources also adds to the strength of the findings. A general caveat to consider when conducting neuroimaging analysis of individuals with DS is that when normalization is required, as in this study, image registration performance may be reduced, as brain structure in DS is thought to be atypical. However, in this case, we used SPM DARTEL to perform highly accurate nonlinear registrations (Klein et al., 2009) and if registration error had been driving the brain-PAD results, then this would increase noise and reduce the sensitivity to relate brain-PAD to external measures such as CAMCOG score. One perhaps surprising result is that the local controls in the study showed significantly “younger” appearing brains, according to brain-PAD scores, relative to the independent training sample. This could potentially be driven by scanner effects, although by design the training sample includes individuals from a range of different MRI scanner systems and field strengths to dilute any potential scanner biases that might cause overfitting and reduce generalizability. Another explanation could be a recruitment bias in that the local controls were comprised of individuals preferentially exposed to positive influences on apparent brain aging, although steps were taken to recruit a wide spectrum of controls from beyond the confines of the University of Cambridge. We were unable to acquire detailed demographic or behavioral data on these individuals, so we could not ascertain whether the variance in brain-PAD controls is related to any potential protective effects, such as increased years of education or physical exercise. Finally, as mentioned above, the cross-sectional nature of this study means that we cannot examine trajectories of change in people with DS, which would provide important information about the likelihood of future neurologic decline and negative brain aging.

## 5 Conclusions

This multimodality neuroimaging study of DS indicates that a consequence of trisomy 21 is an increase in structural brain aging, detectable in middle adulthood. While longitudinal studies are necessary to determine whether this increase in apparent brain aging is static or accelerating, it is notable that the presence of PiB-binding was related to increased brain-PAD and that in DS participants with evidence of beta amyloid deposition, brain-PAD related to cognitive decline. It seems that some people with DS begin to show converging signs of potentially pathologic deterioration, and that this can be detected using T1-weighted MRI to index individual differences in brain aging. When no evidence of PiB binding was observed, both brain aging and cognitive performance remained unaffected. This could imply that amyloid deposition, or related latent factor, could be driving the deleterious brain changes associated with aging in DS.

## Disclosure statement

The authors have no actual or potential conflicts of interest.
