# Supplementary material for: Brain-predicted age in Down syndrome is associated with beta amyloid deposition and cognitive decline
Source: Neurobiol Aging. 2017 Aug;56:41–9. doi: 10.1016/j.neurobiolaging.2017.04.006 (PMC5476346; doi:10.1016/j.neurobiolaging.2017.04.006)
Supplement: Supplementary Tables 1 and 2 [file mmc1.docx]

## Supplementary material

## Supplementary Table 1. Data sources for healthy brain age training sample

| Cohort | N | Age  mean (SD) | Age  range | Sex  male/female | Repository details | Scanner  (Field strength) | Scan | Voxel dimensions |
| --- | --- | --- | --- | --- | --- | --- | --- | --- |
| ABIDE (Autism Brain Imaging Data Exchange) | 184 | 25.93 (6.66) | 18-48 | 161/23 | INDI | Various (all 3T) | MPRAGE | Various |
| Beijing Normal University | 179 | 21.25 (1.92) | 18-28 | 72/107 | INDI | Siemens (3T) | MPRAGE | 1.33x1.0x1.0 |
| Berlin School of Brain & Mind | 49 | 30.99 (7.08) | 20-60 | 24/25 | INDI | Siemens Tim Trio (3T) | MPRAGE | 1.0x1.0x1.0 |
| CADDementia | 12 | 62.33 (6.26) | 55-79 | 9/3 | http://caddementia.grand-challenge.org | GE Signa (3T) | 3D IR-FSPGR | 0.9x0.9x1.0 |
| Cleveland Clinic | 31 | 43.55 (11.14) | 24-60 | 11/20 | INDI | Siemens Tim Trio (3T) | MPRAGE | 2.0x1.0x1.2 |
| ICBM (International Consortium for Brain Mapping) | 322 | 24.84 (5.14) | 24-60 | 177/145 | LONI IDA | Siemens Magnetom (1.5T) | MPRAGE | 1.0x1.0x1.0 |
| IXI (Information eXtraction from Images) | 561 | 48.62 (16.49) | 20-86 | 250/311 | http://biomedic.doc.ic.ac.uk/brain-development | Philips Intera (3T); Philips Gyroscan Intera (1.5T); GE Signa (1.5T) | T1-FFE; MPRAGE | 0.9375x0.93751x1.2 |
| MCIC (MIND Clinical Imaging Consortium) | 93 | 32.49 (11.95) | 18-60 | 64/29 | COINS | Siemens Sonata/Trio (1.5/3T); GE Signa (1.5T) | MPRAGE; SPGR | 0.625x0.625x1.5 |
| MIRIAD (Minimal Interval Resonance Imaging in Alzheimer's Disease) | 23 | 69.66 (7.18) | 58-85 | 12/11 | https://www.ucl.ac.uk/drc/research/miriad-scan-database | GE Signa (1.5T) | 3D IR-FSPGR | 0.9375x0.93751x1.5 |
| NEO2012 (Adelstein, 2011) | 39 | 29.59 (8.38) | 20-49 | 18/21 | INDI | Siemens Allegra (3T) | MPRAGE | 1.0x1.0x1.0 |
| Nathan Kline Institute (NKI) / Rockland | 160 | 41.49 (18.08) | 18-85 | 96/64 | INDI | Siemens Tim Trio (3T) | MPRAGE | 1.0x1.0x1.0 |
| OASIS (Open Access Series of Imaging Studies) | 288 | 44.06 (23.04) | 18-90 | 106/188 | http://www.oasis-brains.org/ | Siemens Vision (1.5T)* | MPRAGE | 1.0x1.0x1.25 |
| WUSL (Power, 2012) | 24 | 23.04 (1.42) | 20-24 | 4/20 | INDI | Siemens Tim Trio (3T) | MPRAGE | 1.0x1.0x1.0 |
| TRAIN-39 | 36 | 22.67 (2.56) | 18-28 | 11/25 | INDI | Siemens Allegra (3T) | MPRAGE | 1.33x1.33x1.3 |
| Training set total | **2001** | **36.95 (18.12)** | **18-90** | **1016/985** | **-** | **-** | **-** | **-** |
| INDI = International Neuroimaging Data-sharing Initiative (<http://fcon_1000.projects.nitrc.org>)  COINS = Collaborative Informatics and Neuroimaging Suite ([http://coins.mrn.org](http://coins.mrn.org/))  LONI = Laboratory of Neuro Imaging Image & Data Archive (<https://ida.loni.usc.edu)>  ABIDE consortiums comprising data from various sites with different scanners/parameters  *OASIS scans were acquired four times and then averaged to increase signal-to-noise ratio. | | | | | | | | |

## Supplementary Table 2. Comparing brain-PAD based on PIB-status and CAMDEX classification

| **PIB-status** | **CAMDEX rating** | **N** | **Brain-PAD (years)** |
| --- | --- | --- | --- |
| PIB-negative | CAMDEX stable | 24 | 0.67 (7.24) |
| PIB-positive |  | 7 | 7.20 (8.00) |
| PIB-negative | CAMDEX declining/dementia | 3 | -0.63 (1.33) |
| PIB-positive |  | 12 | 4.18 (10.31) |
| **Brain-PAD = brain-predicted age difference. Brain-PAD values are reported mean (standard deviation).** | | | |
